# An Immunohistochemical Analysis of Tissue Thrombin Expression in the Human Atria

**DOI:** 10.1371/journal.pone.0065817

**Published:** 2013-06-13

**Authors:** Keiichi Ito, Taro Date, Masahiro Ikegami, Kenichi Hongo, Masami Fujisaki, Daisuke Katoh, Takuya Yoshino, Ryuko Anzawa, Tomohisa Nagoshi, Seigo Yamashita, Keiichi Inada, Seiichiro Matsuo, Teiichi Yamane, Michihiro Yoshimura

**Affiliations:** 1 Division of Cardiology, Department of Internal Medicine, The Jikei University School of Medicine, Minato-ku, Tokyo, Japan; 2 Department of Pathology, The Jikei University School of Medicine, Minato-ku, Tokyo, Japan; University of Kansas Medical Center, United States of America

## Abstract

**Objective:**

Thrombin, the final coagulation product of the coagulation cascade, has been demonstrated to have many physiological effects, including pro-fibrotic actions via protease-activated receptor (PAR)-1. Recent investigations have demonstrated that activation of the cardiac local coagulation system was associated with atrial fibrillation. However, the distribution of thrombin in the heart, especially difference between the atria and the ventricle, remains to be clarified. We herein investigated the expression of thrombin and other related proteins, as well as tissue fibrosis, in the human left atria and left ventricle.

**Methods:**

We examined the expression of thrombin and other related molecules in the autopsied hearts of patients with and without atrial fibrillation. An immunohistochemical analysis was performed in the left atria and the left ventricle.

**Results:**

The thrombin was immunohistologically detected in both the left atria and the left ventricles. Other than in the myocardium, the expression of thrombin was observed in the endocardium and the subendocardium of the left atrium. Thrombin was more highly expressed in the left atrium compared to the left ventricle, which was concomitant with more tissue fibrosis and inflammation, as detected by CD68 expression, in the left atrium. We also confirmed the expression of prothrombin in the left atrium. The expression of PAR-1 was observed in the endocardium, subendocardium and myocardium in the left atrium. In patients with atrial fibrillation, strong thrombin expression was observed in the left atrium.

**Conclusions:**

The strong expression levels of thrombin, prothrombin and PAR-1 were demonstrated in the atrial tissues of human autopsied hearts.

## Introduction

Thrombin, the final coagulation product of the coagulation cascade, plays various physiological roles, including pro-fibrotic actions via protease-activated receptor (PAR)-1, PAR-2 and PAR-4 [1]. PAR-1 is also involved in vessel wound healing and revascularization [2], platelet procoagulant activity [3] and gastric contraction [4]. In lung tissue, the induction of myofibroblasts occurs primarily via the actions of PAR-1 [5], [6], and a recent study demonstrated the importance of PAR-1 in the pathogenesis of fibrosis in cardiac fibroblasts [7].

Recent clinical investigations have demonstrated that the local coagulation system in the heart is activated in patients with atrial fibrillation [8], [9], [10]. Thrombin is known to exist in several tissues, such as the endothelium [11] and fibroblasts [12]. However, there have been few reports that have immunohistologically analyzed the distribution of thrombin in the heart, or the roles of tissue thrombin in the inflammatory process and fibrosis, which is a substrate of atrial tachyarrhythmias [13].

In this study, we investigated the expression of thrombin and other related molecules in the left atrium and left ventricle of patients with and without atrial fibrillation.

## Methods

Informed consent have been obtained and all clinical investigation have been conducted according to the principles expressed in the Declaration of Helsinki. We have obtained approval from the Ethics Committee of Jikei University School of Medicine. We did not conduct research outside of our country of residence. The full names of our ethics committees and the institutions/hospitals we are associated with are “the Ethics Committee of Jikei University School of Medicine”. Participants have provided their written informed consent to participate in this study. An immunohistochemical analysis of the expression and localization of thrombin, prothrombin, PAR-1 and CD68 was performed in 7 patients (patient 1: a 71-year-old male who died of ischemic colitis and septic shock and had no history of atrial fibrillation, patient 2: an 85-year-old male who died of hepatocellular carcinoma caused by hepatitis C virus infection and had no history of atrial fibrillation, patient 3: a 67-year-old male who died of chronic lymphocytic lymphoma and had no history of atrial fibrillation, patient 4: a 77-year-old male who died of pneumonia and had no history of atrial fibrillation, patient 5: a 50-year-old male who died of acute myeloid leukemia and had no history of atrial fibrillation, patient 6: a 75-year-old male who died of intrahepatic bile duct carcinoma who had a history of paroxysmal atrial fibrillation, patient 7: a 69-year-old male who died of pneumonia and had a history of ventricular tachycardia and atrial fibrillation).

Sections obtained from formalin-fixed, paraffin-embedded specimens were stained with hematoxylin and eosin and Masson trichrome stain. For the immunohistochemical analyses, sections were deparaffinized and digested with 0.05% subtilisin. The inactivation of endogenous peroxidase activity was performed by incubation in 3% H_2_O_2_ in methanol for 30 minutes. After several washes in phosphate buffered saline (PBS), the slides were heated in a microwave oven at 121°C for antigen retrieval. After being cooled at room temperature and washed with PBS, the sections were incubated with blocking solution for one hour at room temperature. Then, after PBS washing, the tissues were bordered with a pap-pen. The sections were incubated with mouse monoclonal antibodies against thrombin (Santa Cruz, Delaware Avenue, CA), PAR-1 (Santa Cruz, Delaware Avenue, CA), PAR-2 (Santa Cruz, Delaware Avenue, CA), PAR-4 (Santa Cruz, Delaware Avenue, CA), alpha-smooth muscle actin (αSMA) (Dako, Carpenteria, CA) and CD68 (Dako, Carpenteria, CA), or with rabbit polyclonal antibodies against prothrombin (Bioworld Technology, England) and PAR-3 (Santa Cruz, Delaware Avenue, CA) for 30 minutes at room temperature following standard protocols. The antigen retrieval was changed based on the primary antibodies used. For the thrombin and PAR-4 antibodies, antigen retrieval was performed in citric acid buffer (pH 6.0, 0.01 M) for 10 minutes. For the PAR-1 antibody, antigen retrieval was performed in target retrieval solution with a high pH (Dako, Carpenteria, CA) for 10 minutes. For the prothrombin and CD68 antibodies, antigen retrieval was carried out in protease for one minute. The sections were visualized using Nikon Eclipse 80i with a Nikon Digital Camera DXM 1200. CD68-positive cells were enumerated using a ×40 objective lens as described previously [14]. The fields were chosen at random by blind and sequential movement of the mechanical stage. Very large vessels were excluded from the counts. At least 20 random fields were counted. The immunohistochemical staining was scored subjectively on a semi-quantitative scale of 0–4 (0 =  no staining, 1 =  weak staining, 2 =  moderate staining, 3 =  strong staining, 4 =  intense staining), as described previously [15]. Statistical comparisons between groups were performed using the Wilcoxon test. All statistical analyses were performed using the SPSS software program (version 21, SPSS Japan Inc., Tokyo, Japan), and differences were considered to be statistically significant for values of p<0.05.

## Results

**Figure 1:**
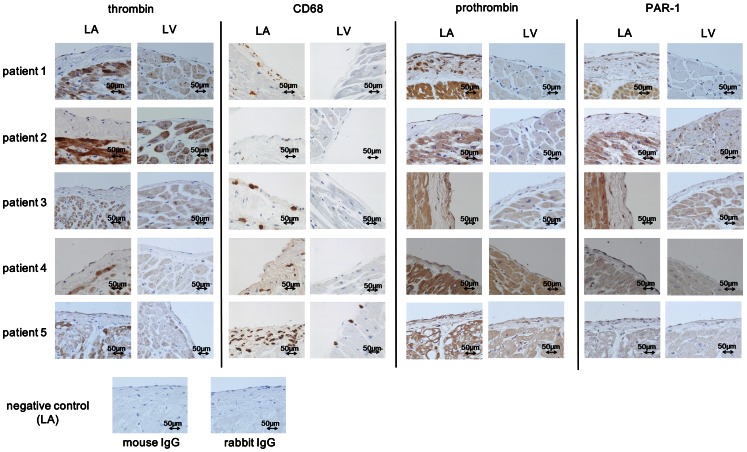
The results of the immunohistochemical analysis of the expression of thrombin, CD68, prothrombin and PAR-1 in the LA and the LV around the endocardium obtained from human autopsied hearts of patients without any history of AF (×40). The expression of thrombin, CD68, prothrombin and PAR-1 was evident in the LA around the thick subendocardial space of the LA. LA: left atrium, LV: left ventricle.

**Figure 2:**
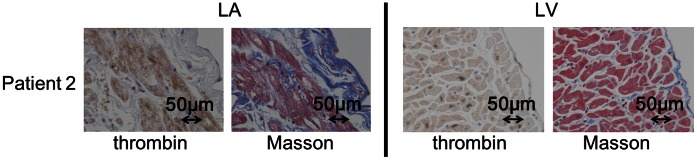
The results of the immunohistochemical analysis of the thrombin expression and Masson trichrome staining in the serial sections obtained from patient (×10). LA: left atrium, LV: left ventricle. A larger fibrotic area was observed in the LA than in the LV.

The immunohistochemical analysis demonstrated the expression of tissue thrombin to be observed in the endocardium, subendocardium and myocardium in the left atrium (LA) and the left ventricle (LV) in all five patients without a history of AF (Figure 1). In the myocardium of the LA, as in the endocardium, the thrombin expression levels were higher in the LA compared to the LV (Figure 1, averaged scores: 2.4 vs. 0.8 in the endocardium/subendocardium, 2.6 vs. 1.0 in the myocardium, p<0.05). The CD68 positivity was also more prominent in the endocardium/subendocardium of the LA than the LV (Figure 1, 5.9/high power field (LA) vs. 3.7/high power field (LV), p<0.05). Masson trichrome staining showed that more fibrosis was present in the subendocardial space and interstitium of the LA compared to that of the LV, which was concomitant with the thrombin expression (Figure 2).

**Figure 3:**
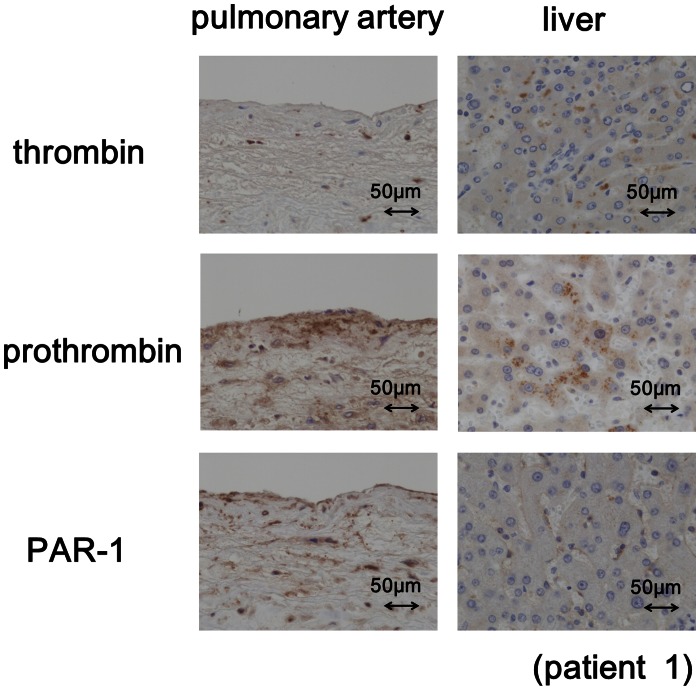
The results of an immunohistochemical analysis of the expression of thrombin, prothrombin and PAR-1 in the pulmonary artery and liver of human autopsied tissues. The expression of thrombin, prothrombin and PAR-1 was confirmed in the liver (positive control). Thrombin was sparsely stained, and both prothrombin and PAR-1 were distinctly detected in the pulmonary artery.

We also investigated the expression of prothrombin and PAR-1 in the LA and LV, and found that they were both detected in the endocardium, subendocardium and myocardium of the LA in all of the studied patients without a history of AF (Figure 1). The semi-quantitative scores of the prothrombin expression were higher in the LA compared to the LV (Figure 1, averaged scores: 3.4 vs. 1.0 in the endocardium/subendocardium, 2.8 vs. 1.2 in the myocardium, p<0.05). The PAR-1 expression was also stronger in the LA compared to the LV (Figure 1, averaged scores: 2.4 vs. 0.8 in the endocardium/subendocardium, 2.2 vs. 0.8 in the myocardium, p<0.05). We also examined the expression levels of these molecules in tissue specimens from other organs, and significant thrombin, prothrombin and PAR-1 expression was detected in the liver, which also served as a positive control for prothrombin expression, and in the pulmonary artery wall (Figure 3).

**Figure 4:**
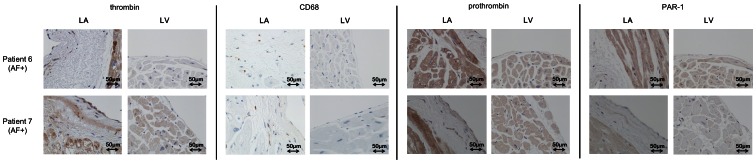
The results of an immunohistochemical analysis of the expression of thrombin, CD68, prothrombin and PAR-1 in the LA of autopsied hearts from patients with AF (AF(+): patient 6 and patient 7) (×40). Thrombin was highly expressed in the myofibers, as well as the endocardium and subendocardium. CD68 positivity was noted in the LA, but was comparable to that in the patients without AF. Prothrombin and PAR-1 were highly detected in these sections in the LA. LA: left atrium.

**Figure 5:**
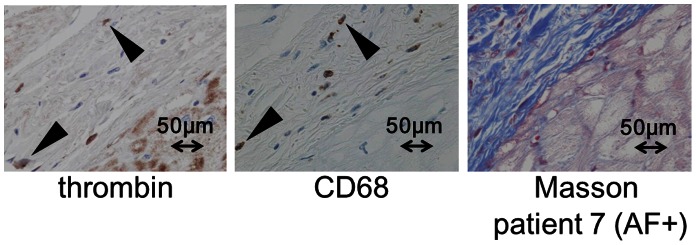
The results of an immunohistochemical analysis of the expression of thrombin and CD68, as well as Masson trichrome staining, in the serial sections of the LA from the autopsied heart of a patient with AF (patient 7). Notably, the co-localization of the CD68-stained areas with the thrombin expression was revealed, indicating that the source of thrombin could be invasive macrophages present in the atria. A thick fibrotic sub-endocardial layer was present in the LA, as indicated by Masson trichrome staining. Masson: Masson trichrome staining, LA: left atrium, AF: atrial fibrillation.

**Figure 6:**
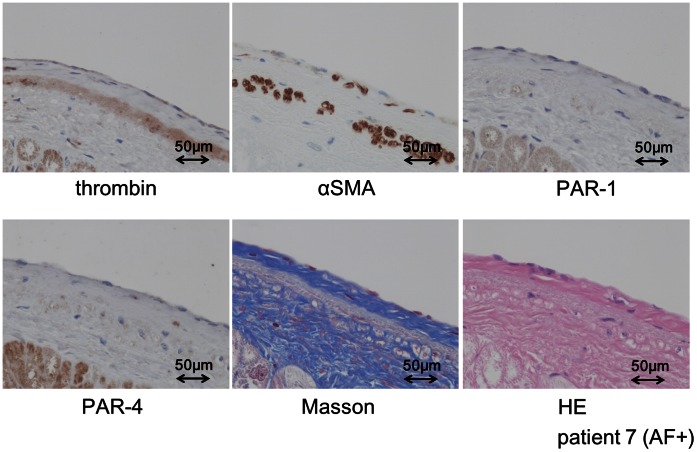
The results of an immunohistochemical analysis of the expression of thrombin, αSMA, PAR-1 and PAR-4, as well as Masson trichrome staining and HE staining, in the serial sections of the LA from the autopsied heart of a patient with AF (patient 7). Co-localization of the αSMA-stained areas with the thrombin expression was noted in the fibrotic sub-endocardial layer of the LA, as determined by Masson trichrome staining. Masson: Masson trichrome staining, LA: left atrium, AF: atrial fibrillation.

We next examined the expression of thrombin and other related molecules in patients with atrial fibrillation. An immunohistological analysis of the LA from patients with AF showed strong thrombin expression in the myocardium and thick, fibrotic subendocardial space of the LA (averaged scores: 3.0 in the endocardium/subendocardium, 3.5 in the myocardium) (Figure 4) as well as strong prothrombin expression (averaged scores: 3.5 in the endocardium/subendocardium and 4 in the myocardium) and PAR-1 expression (averaged scores: 3.5 in the endocardium/subendocardium and 2.5 in the myocardium) (Figure 4). The tissue thrombin detected in the subendocardial space of LA was co-localized with CD68-positive areas, indicating that some of the thrombin was derived from macrophages (Figure 5). PAR-4, another important thrombin receptor, was positively stained in the LA (Figure 6), whereas PAR-2 and PAR-3 expression were barely observed in the LA (data not shown). Of note, some of the tissue thrombin in the subendocardial space of the LA was co-localized with the αSMA expression, which is a marker of the profibrogenic myofibroblast phenotype (Figure 6). These findings suggest that thrombin plays an important role in promoting atrial fibrosis through the conversion of cardiac fibroblasts to a profibrogenic phenotype.

## Discussion

The present study demonstrated, for the first time, that the prominent local expression of thrombin can be detected immunohistochemically in the human atrium, and that this expression was partially associated with the CD68 expression and tissue fibrosis.

Recent data suggest the possible involvement of the coagulation system in various fibrotic diseases, including pulmonary fibrosis. The process of activating the coagulation system depends on the actions of thrombin. There are four types of thrombin receptors: PAR-1, PAR-2, PAR-3 and PAR-4. These thrombin receptors are members of the G-protein-coupled receptor (GPCR) family [16]. Previous studies have reported that the various physiological roles of thrombin are mediated through the actions of PAR-1 in cardiac fibroblasts^7)^. Thrombin interacts with PAR-1, PAR-2 and PAR-4, and cleaves their N-terminus to unveil the pentapeptide [16]. The existence of thrombin in several tissues has been suggested in previous reports [11], [12], [16]. Akar et al. demonstrated that activation of the cardiac local coagulation system was associated with paroxysmal attacks of atrial fibrillation [9]. These previous investigations prompted us to immunohistologically analyze the expression levels of thrombin and related molecules in the human heart. In the present study, we investigated the expression levels of thrombin and prothrombin in the LA, in which we were able to detect both thrombin and prothrombin in the endocardium, subendocardium and myocardium. In addition, our data provide evidence that PAR-1 and PAR-4 are expressed in the atrial tissue, thus suggesting that thrombin plays an important functional role in the atria. Notably, the co-localization of the CD68-stained areas with the expression of prothrombin and thrombin revealed that the source of thrombin could be invasive macrophages present in the atrium. Yamashita et al. demonstrated active adhesion and recruitment of macrophages across the endocardium in human fibrillating atria, with local inflammatory responses around the endocardial regions [17]. Our observations prompted us to hypothesize that the inflammation and the subsequent fibrotic changes induced by invasive macrophages around the atrial endocardium may be at least partly mediated by thrombin. The present study demonstrated that increased thrombin expression was observed in the rich fibrotic areas, especially in the LAs of the patients with a history of AF. A recent investigation demonstrated that thrombin induced the conversion of cardiac fibroblasts to a profibrogenic myofibroblast phenotype, as indicated by αSMA expression, via PAR-1 activation and an increase in collagen synthesis [7]. Our present observations demonstrated that αSMA was co-localized with the thrombin expression, especially in the sub-endocardial region. Taken together, these findings suggest that tissue thrombin in the atria may promote atrial fibrosis, which can cause atrial tachyarrhythmias. Recent findings have shown that PAR-1 and PAR-4 activation both contribute to cardiac remodeling and influence cardiac inflammation [18], [19]. PAR-1 and PAR-4 signaling might also be related to tissue fibrosis in the atria. The precise reason why increased thrombin expression was observed in the LA of the patients with a history of AF remains to be determined. However, it was reported that oxidant stress may enhance the thrombin expression in brain endothelial cells [20]. It is therefore possible that elevated oxidative stress, which is associated with the pathogenesis of AF [21], may lead to the upregulation of thrombin expression. Further studies are required to determine the physiological and pathological roles of tissue thrombin.

The present study demonstrated the staining for thrombin in the human atria tissue. Two mechanisms; the expression and the clearance, were estimated to underlie the staining of thrombin. In the endothelium, thrombin co-localizes with thrombomodulin, and they are both internalized and removed from the circulation by the endothelium [11]. The clearance of thrombin from the circulation occurs via two pathways [22]. One pathway involves the endothelial route discussed above, and the other involves its removal by the liver. Antithrombin III binds to thrombin, and the thrombin-antithrombin III complex is removed by the liver [22]. Therefore, the endothelium and liver both clear thrombin from the circulation. Although the presence of the tissue thrombin outside of the endothelium has been reported in chick embryonic fibroblasts [12], the role of tissue thrombin has not been elucidated [12]. The presence of prothrombin in tissues has also been reported in a previous study [23]. Functionally intact prothrombin is widely distributed among various tissues, including the myocardium; however, the physiological significance of tissue prothrombin remains unclear [23]. Although the thrombin of the atrial tissue may be derived from the cleared thrombin from the circulation across the endocardium and the vascular endothelium, the possibility of local production of thrombin in the atria cannot be ruled out.

In conclusion, the expression of thrombin in the atrial tissues of human autopsied hearts was demonstrated. Further research is needed to determine the physiological and pathological roles of atrial tissue thrombin.
